# The *Acinetobacter baumannii entA* Gene Located Outside the Acinetobactin Cluster Is Critical for Siderophore Production, Iron Acquisition and Virulence

**DOI:** 10.1371/journal.pone.0036493

**Published:** 2012-05-03

**Authors:** William F. Penwell, Brock A. Arivett, Luis A. Actis

**Affiliations:** Department of Microbiology, Miami University, Oxford, Ohio, United States of America; Vrije Universiteit Brussel, Belgium

## Abstract

*Acinetobacter baumannii* causes severe infections in compromised patients, who present an iron-limited environment that controls bacterial growth. This pathogen has responded to this restriction by expressing high-affinity iron acquisition systems including that mediated by the siderophore acinetobactin. Gene cloning, functional assays and biochemical tests showed that the *A. baumannii* genome contains a single functional copy of an *entA* ortholog. This gene, which is essential for the biosynthesis of the acinetobactin precursor 2,3-dihydroxybenzoic acid (DHBA), locates outside of the acinetobactin gene cluster, which otherwise harbors all genes needed for acinetobactin biosynthesis, export and transport. *In silico* analyses and genetic complementation tests showed that *entA* locates next to an *entB* ortholog, which codes for a putative protein that contains the isochorismatase lyase domain, which is needed for DHBA biosynthesis from isochorismic acid, but lacks the aryl carrier protein domain, which is needed for tethering activated DHBA and completion of siderophore biosynthesis. Thus, *basF*, which locates within the acinetobactin gene cluster, is the only fully functional *entB* ortholog present in ATCC 19606^T^. The differences in amino acid length and sequences between these two EntB orthologs and the differences in the genetic context within which the *entA* and *entB* genes are found in different *A. baumannii* isolates indicate that they were acquired from different sources by horizontal transfer. Interestingly, the AYE strain proved to be a natural *entA* mutant capable of acquiring iron via an uncharacterized siderophore-mediated system, an observation that underlines the ability of different *A. baumannii* isolates to acquire iron using different systems. Finally, experimental infections using *in vivo* and *ex vivo* models demonstrate the role of DHBA and acinetobactin intermediates in the virulence of the ATCC 19606^T^ cells, although to a lesser extent when compared to the responses obtained with bacteria producing and using fully matured acinetobactin to acquire iron.

## Introduction

*Acinetobacter baumannii* is being increasingly recognized as an important pathogen that causes severe infections in hospitalized patients as well as deadly cases of community-acquired pneumonia [1], [2], [3], [4]. More recently, it has been described as the ethiological agent of severe wound infections in military personnel injured in the Middle East [5], [6] and cases of necrotizing fasciitis [7]. A serious concern with this pathogen is its remarkable ability to acquire genes and express resistance to a wide range of antibiotics as well as to evade the human defense responses [4]. Among the latter is the capacity of *A. baumannii* to prosper under the iron-limited conditions imposed by the human host's high-affinity chelators lactoferrin and transferrin [8], [9]. Although some progress has been made in recent years, not much is known about the pathobiology of this bacterium and the nature of its virulence factors involved in the serious diseases it causes in humans.

Bacterial pathogens respond to iron limitation imposed by the human host by expressing different high-affinity uptake systems including siderophore-dependent and siderophore-independent systems, as well as systems that remove iron from host compounds, such as hemin, by either direct contact or by producing scavengers known as hemophores [10], [11]. In the case of *A. baumannii*, experimental data [12], [13], [14], [15] and *in silico* analyses of fully sequenced and annotated genomes [16], [17] show that different *A. baumannii* clinical isolates could express different iron uptake systems. Currently, the best-characterized system is that expressed by the ATCC 19606^T^ type strain, which is based on the production and utilization of acinetobactin [14], [18], [19]. This catechol-hydroxamate siderophore is a non-cyclic derivative of 2,3-dihydroxybenzoic acid (DHBA) linked to threonine and *N*-hydroxyhistamine [19]. Genetic and functional analyses indicate that the acinetobactin-mediated system is the only high-affinity iron acquisition system expressed by the ATCC 19606^T^ type strain [14]. The *bas*, *bau* and *bar* genes needed for the production, transport and secretion of acinetobactin, respectively, are located in a 26.5-kb chromosomal region harboring seven operons [14], [18]. However, this locus does not include an *entA* ortholog coding for a 2,3-dihydro-2,3-dihydroxy-benzoate dehydrogenase. This enzyme is involved in the last step of the conversion of chorismate into DHBA, which is essential for the biosynthesis of the catechol moiety of siderophores such as enterobactin [20], [21]. This observation indicates that at least two chromosomal regions are involved in the biosynthesis of acinetobactin in the ATCC 19606^T^ strain; one containing the *bas*, *bau* and *bar* genes and another harboring at least the *entA* gene. In this report, we present experimental and genomic evidence supporting this hypothesis as well as showing that there are variations not only in nucleotide sequence but also in genetic arrangements among the *A. baumannii* loci harboring the *entA* genetic determinant. In addition, we demonstrate that the expression of an active *entA* gene is needed for the full virulence of the ATCC 19606^T^ strain when tested using A549 human alveolar epithelial cells and *Galleria mellonella* caterpillars as experimental infection models. We also report the observation that the *A. baumannii* AYE strain is a natural *entA* mutant that acquires iron through a siderophore-mediated system that remains to be characterized.

## Materials and Methods

### Bacterial strains, plasmids, and culture conditions

Bacterial strains and plasmids used in this work are shown in Table S1. Strains were routinely cultured in Luria Bertani (LB) broth or agar [22] at 37°C in the presence of appropriate antibiotics. Iron-rich and iron-limiting conditions were achieved by the addition of FeCl_3_ dissolved in 0.01 M HCl and 2,2′ dipyridyl (DIP), respectively, to liquid or solid media.

### Recombinant DNA techniques

Chromosomal and plasmid DNA were isolated by ultracentrifugation in CsCl density gradients [22], [23] or using commercial kits (Qiagen). DNA restriction and Southern blot analyses were conducted using standard protocols and [^32^P]α-dCTP-labelled probes prepared as described before [22], [24].

### Construction of a gene library and cloning of the *entA* gene

**Figure 1:**
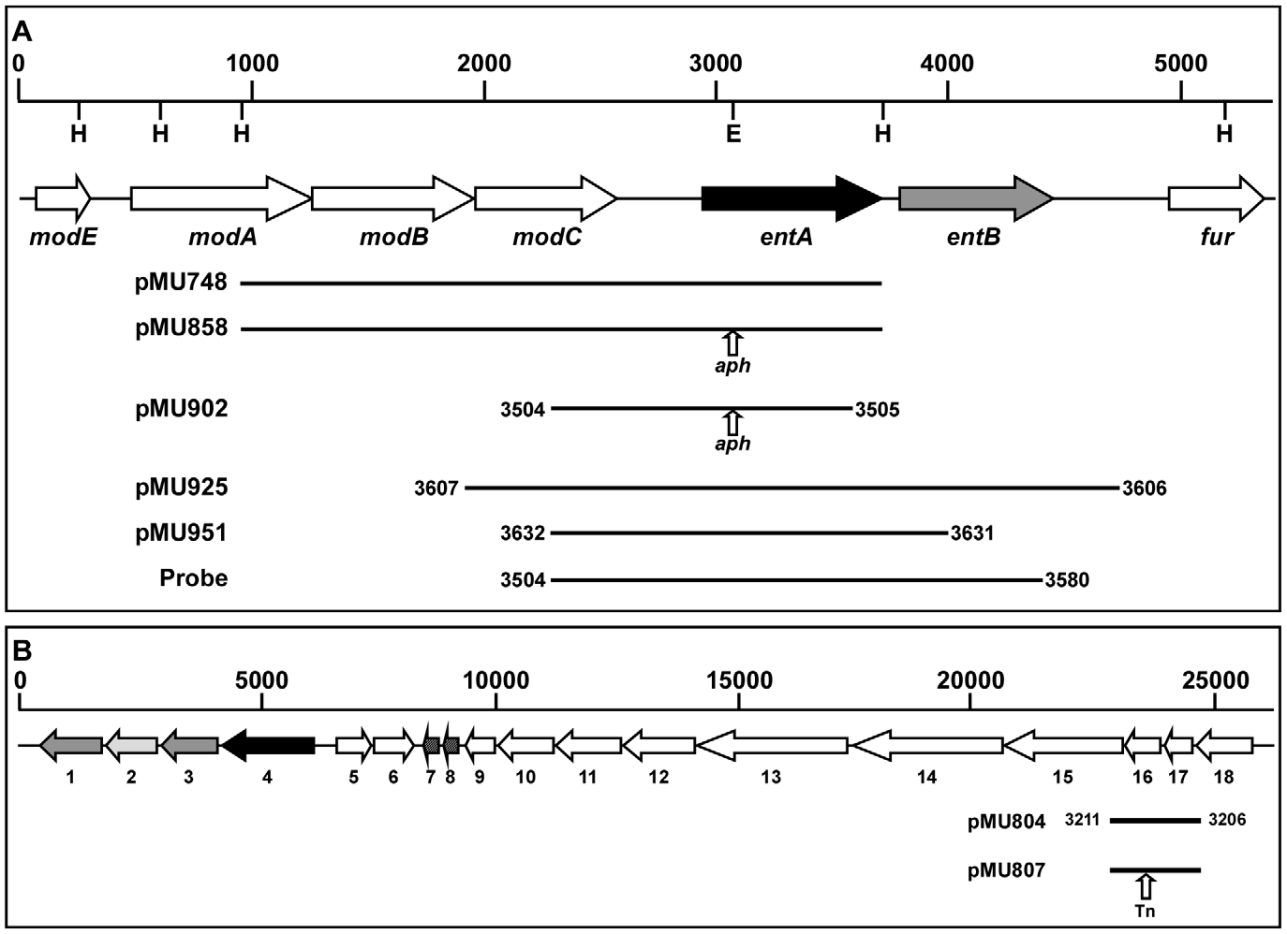
Genetic organization of *A. baumannii* chromosomal regions harboring genes coding for siderophore production and utilization. (A) Genetic map of the ATCC 19606^T^ gene cluster containing the *entA* and *entB* orthologs. H, HindIII; E, EcoRI. (B) Genetic map of the ATCC 17978 gene cluster that includes the predicted *entA* and *entB* orthologs. The horizontal arrows and their direction indicate the location and direction of transcription of predicted genes, respectively. The horizontal lines indicate the chromosomal regions either cloned or used to confirm recombinant derivatives by PCR or Southern blotting. The numbers next to each bar indicate the location of primers used in PCR amplification. The vertical arrows indicate the insertion of the DNA cassette harboring the *aph* gene, which code for kanamycin resistance, or the EZ-Tn*5*<KAN-2> transposon (Tn). Numbers under ORFs shown in panel B correspond to those listed in Table 2. Numbers on top of short vertical bars indicate DNA size in kb.

An *A. baumannii* ATCC 19606^T^ genomic library was prepared using *Escherichia coli* LE392 and the cosmid vector pVK100 as described before [13]. Cosmid DNA was isolated *en masse* from *E. coli* LE392 clones and used to transform *E. coli* AN193 by electroporation as described before [13]. Transformants harboring the ATCC 19606^T^ *entA* gene were selected on LB agar containing 20 µg/ml tetracycline (Tet) and 250 µM DIP. Cosmid DNA was isolated from one of the *E. coli* AN193 complemented clones, which was named 2631, digested with HindIII and subcloned into pUC118 to generate pMU748 (Fig. 1A). Plasmid DNA was isolated from *E. coli* DH5α recombinant subclones and sequenced with standard automated DNA sequencing methods using M13 forward and reverse [25] and custom-designed primers. Sequences were assembled using Sequencher 4.2 (Gene Codes Corp.). Nucleotide and amino acid sequences were analyzed with DNASTAR, BLAST [26], and the software available through the ExPASy Molecular Biology Server (http://www.expasy.ch).

### Construction of an ATCC 19606^T^ *entA*::*aph* isogenic derivative

To generate the ATCC 19606^T^ *entA*::*aph* insertion mutant 3069, a 2.5-kb pMU858 fragment, which encompasses the pUC4K DNA cassette inserted into an EcoRV site located within *entA*, was PCR amplified with primers 3504 (5′-CCAACAAGAACGTCACTT-3′) and 3505 (5′-ATTCCTGTTCGGTACTGG-3′) (Fig. 1A) and Phusion DNA polymerase (NEB). The amplicon was cloned into the SmaI site of the pEX100T and *E. coli* DH5α transformants were selected on LB agar containing 40 µg/ml kanamycin (Km) and 150 µg/ml ampicillin (Amp). Plasmid DNA (pMU902) was isolated from one of these derivatives and the appropriate cloning was confirmed by automated sequencing using primers 3187 (5′-AGGCTGCGCAACTGTTGG-3′) and 3188 (5′-TTAGCTCACTCATTAGGC-3′), which anneal close to the pEX100T SmaI site. ATCC 19606^T^ cells were electroporated with pMU902 as described before [27] recovering the cells in SOC medium [22] for 6 h in a shaking incubator at 37°C. Transformants were selected on LB agar containing 40 µg/ml Km. The generation of the appropriate ATCC 19606^T^ *entA*::*aph* derivative was confirmed by PCR using primers 3504 and 3580 (5′-CCATGCTTGGATTACTTG-3′) (Fig. 1A) as well as Southern blotting [22] using as a probe the amplicon obtained with primers 3504 and 3580 (Fig. 1A) and parental DNA as a template. The ATCC 19606^T^ 3069 derivative was genetically complemented with pMU951 (Fig. 1A), a derivative of the shuttle vector pWH1266 harboring an amplicon encompassing the parental *entA* allele that was obtained with Phusion DNA polymerase using primers 3631 (5′-GGATCCGGGAATATTAGACTGGCG-3′) and 3632 (5′-GGATCCCCAACAAGAACGTCACTT-3′), both of which included BamHI restriction sites.

### Production and utilization of catechol and siderophore compounds and expression of EntA and EntB activity by cloned genes

*A. baumannii* cells were cultured in a chemically defined medium containing sodium succinate as a carbon source [19]. Production of extracellular compounds with siderophore activity was investigated with the Chrome Azurol S (CAS) reagent [28]. The presence of catechol compounds in cell-free succinate culture supernatants [19] was detected with the Arnow test [29]. Briefly, 1 vol of reagent A (0.5 N HCl), reagent B (10% Na nitrite, Na molybdate), and reagent C (1 N NaOH) were successively added to 1 vol of culture supernatant cleared by centrifugation at 16,000× g. The reaction was measure by determining OD_510_ after 10 min incubation at room temperature. DHBA (Sigma-Aldrich) was used as a standard in chemical and biological assays. Production of DHBA was biologically examined with cross-feeding assays using the *Salmonella typhimurium enb*-7 enterobactin mutant as previously described [15]. Minimal inhibitory concentrations (MICs) of DIP, which were repeated at least three times in duplicate each time, were determined using M9 minimal medium [30] containing increasing concentrations of DIP. OD_600_ was used to monitor cell growth after overnight incubation at 37°C. Expression of EntA and EntB activity of cloned DNA was tested by transforming the *E. coli* AN193 and *E. coli* AN192 mutants with pMU925, which was obtained by PCR amplification and cloning of the ATCC 19606^T^ genomic region encompassing the predicted *entA* and *entB* genes with primers 3606 (5′-GAACTGAACCATATGGCG-3′) and 3607 (5′-CGCAGTGGTTTCATCGTT-3′) (Fig. 1A). The same set of primers were used to PCR clone the cognate chromosomal region from the AYE clinical isolate to generate the derivative pMU968. Primers 3206 (5′-CGCAGGCATCGTAAAGGG-3′) and 3211 (5′-TCTGCACAGCATCAACCG-3′) were used to PCR amplify and clone the ATCC 17978 *entA* and *entB* orthologs (pMU804) (Fig. 1B).

The genetic nature of the *entB* deficient phenotype of *E. coli* AN192 was examined by automated DNA sequencing of amplicons obtained using the primers 3783 (5′-CGTGAACAGGGTATTGCC- 3′) and 3785 (5′-CAGCTAACAGTCGCTGAC -3′) and chromosomal DNA obtained form this mutant and the parental strain AB1515 [31] as templates. PCR sequencing reactions were done using primers 3783, 3785, 3784 (5′-CCTTTGAACCGCAACGTG-3′), 3786 (5′-CGATATCACCATGCACTT), 3792 (5′-CACATTGGCTGTATGACC-3′), and 3793 (5′-CTCCAGCGGAGAACGATG-3′).

Production of DHBA and acinetobactin was examined by HPLC analysis with an Agilent 1100 LC instrument using succinate culture supernatants filtered with 0.45 µm cellulose acetate filter units (Spin-x centrifuge filter units, Costar, Cambridge, MA). Supernatants were fractionated with a Vydac C-8, 5 µm, 250 mm×4.6 mm reversed-phase column (Grace Davison Discovery Sciences, Deerfield, IL). Water and acetonitrile containing 0.13% and 0.1% trifluoroacetic acid, respectively, were used as mobile phases. The gradient was as follows: 17% acetonitrile for 5 min and then from 17% to 30% within 30 min, and thereafter held for 15 min. Detection was at 317 nm with a flow rate of 0.5 ml/min.

### A549 infection assays

A549 human alveolar epithelial cells [32], which were provided by Dr. E. Lafontaine (College of Veterinary Medicine, University of Georgia, USA) were cultured and maintained in DMEM supplemented with 10% heat-inactivated fetal bovine serum at 37°C in the presence of 5% CO_2_ as previously described [33]. A549 monolayers maintained in modified Hank's balanced salt solution (mHBSS, same as HBSS but without glucose) for 24 h at 37°C in 5% CO_2_ without infection remained viable as determined by trypan blue exclusion assays. 24-well tissue culture plates were seeded with approximately 10^4^ epithelial cells per well and then incubated for 16 h. Bacterial cells were grown 24 hr in LB broth at 37°C with shaking at 200 rpm, collected by centrifugation at 15,000 rpm for 10 min, washed, resuspended, and diluted in mHBSS. The A549 monolayers were singly infected with 10^3^ cells of the ATCC 19606^T^ parental strain, the s1 (*basD*) or 3069 (*entA*) isogenic derivatives. Inocula were estimated spectrophotometrically at OD_600_ and confirmed by plate count. Infected monolayers were incubated 24 h in mHBSS at 37°C in 5% CO_2_. The tissue culture supernatants were collected, the A549 monolayers were lysed with sterile distilled H_2_O and lysates were added to the cognate tissue culture supernatants. Bacteria were collected from the resulting suspensions by centrifugation, resuspended in 1 ml sterile distilled H_2_O, serially diluted and then plated on nutrient agar. After overnight incubation at 37°C, the colony forming units (CFUs) were counted and CFU/ml values for each sample were calculated and recorded. Counts were compared using the Student's t-test; *P* values<0.05 were considered significant. Experiments were done four times in triplicate using fresh biological samples each time. To determine bacterial relative fitness, the recovered CFUs were divided by the CFUs of the inoculum used to infect monolayers.

### *G. mellonella* killing assays

Bacteria grown in LB broth were collected by centrifugation and suspended in phosphate-buffered saline solution (PBS). The number of bacteria was estimated spectrophotometrically at OD_600_ and diluted in PBS to appropriate concentrations. All bacterial inocula were confirmed by plating serial dilutions on LB agar and determining colony counts after overnight incubation at 37°C. Ten freshly-received final-instar *G. mellonella* larvae (Grubco, Fairfield, OH) weighing 250–350 mg were randomly selected and used in killing assays as described previously [34]. Briefly, the hemocoel at the last left proleg was injected with 5-µl inocula containing 1×10^5^ bacteria ±0.25 log of each tested strain using a syringe pump (New Era Pump Systems, Inc., Wantagh, NY) with a 26 G½ needle. Each test series included control groups of non-injected larvae or larvae injected with sterile PBS or PBS containing 100 µM FeCl_3_. The test groups included larvae infected with the parental strain ATCC 19606^T^, the s1 *basD* mutant or the 3069 *entA* insertion derivative, which were injected in the absence or presence of 100 µM FeCl_3_. Injected larvae were incubated at 37°C in darkness, assessing death at 24-h intervals over six days. Larvae were considered dead and removed if they did not respond to probing. Results were not considered if more than two deaths occurred in the control groups. Experiments were repeated three times using 10 larvae per experimental group and the survival curves were plotted using the Kaplan-Meier method [35]. *P* values<0.05 were considered statistically significant for the log-rank test of survival curves (SAS Institute Inc., Cary, NC).

## Results and Discussion

### The acinetobactin locus in different *A. baumannii* strains

Previous reports described a 26.5-kb *A. baumannii* ATCC 19606^T^ chromosomal gene cluster involved in acinetobactin production and utilization [14], [18]. Table 1 shows that the same *bas-bau-bar* 18-gene cluster, recently referred to as the acinetobactin gene cluster [17], is also found in *A. baumannii* AYE [36]. In contrast, the cognate clusters in strains AB0057 and ACICU include the additional putative genes AB57_2807 and AB57_2818 [37], and ACICU_02575 and ACICU_02586 [38], respectively. Furthermore, the *A. baumannii* AB307-294 *bas-bau-bar* gene cluster has three additional predicted genes; ABBFA_001054, ABBFA_001053, and ABBFA_001065 [37]. It is not clear whether these additional coding units are the result of sequencing and/or annotation artifacts and their potential role in siderophore production and utilization remains to be tested considering that most of them (AB57_2807, AB57_2818, ACICU_02575, ACICU_02586, ABBFA_001054 and ABBFA_001065) code for polypeptides containing 43 to 51 amino acid residues. Similarly, the annotation of the ATCC 17978 *bas-bau-bar* gene cluster [39] encompasses 21 rather than 18 predicted genes because of potential nucleotide sequencing errors (Table 1). The products of coding regions A1S_2382 and A1S_2383 are highly related to the BasD predicted protein, while the products of the A1S_2376-A1S_2378 coding regions highly match that of the *barA* gene originally described in the ATCC 19606^T^ type strain [18]. Unfortunately, these errors were also included in a recent report analyzing potential iron acquisition functions expressed by *A. baumannii* using bioinformatics [17].

**Table 1:** Components of the *bas-bau-bar* gene cluster in different *A. baumannii* genomes.

Strain	Annotated coding regions	Additional coding regions^a^	Accession #
19606^T^	BAC87896-BAC87913	NA^b^	AB101202
AYE	ABAYE1087-ABAYE1104	None	NC_010410
AB0057	AB57_2802-AB57_2822	2807/2818	NC_011586.1
ACICU	ACICU_02570-ACICU_02589	02575/02586	NC_010611.1
AB307-294	ABBFA_001050-ABBFA_001070	001054-001053/001065	NC_011595.1
17978	A1S_2372-A1S_2392	2382-2383^c^/2376-2378^d^	NC_009085.1

a Additional coding regions compared to the ATCC 19606 *bas-bau-bar* gene cluster. Numbers should be preceded by the cognate letters and underscore symbols.

b Not applicable.

c *basD*-related coding sequences.

d *barA*-related coding sequences.

In spite of all these differences, a common feature of the *bas-bau-bar* gene cluster present in all these strains is the absence of an *entA* gene coding for a 2,3-dihydro-2,3-dihydroxybenzoate dehydrogenase needed for the biosynthesis of DHBA, which is a precursor needed for the production of catechol siderophores, such as enterobactin [21]. This observation indicates that a second locus must contain the *entA* ortholog, a possibility that is supported by our initial finding that the genome of the *A. baumannii* ATCC 17978 strain has an additional gene cluster (Table 2) potentially involved in siderophore biosynthesis and utilization [40]. The coding region A1S_2579 of this cluster, which was identified as cluster 2 [17], was annotated as a putative *entA* ortholog [39]. However, initial BLAST searches using A1S_2579 as a query did not identified *entA* orthologs as top matches in any of the fully sequenced and annotated *A. baumannii* genomes and the ATCC 19606^T^ partial genomic data (GenBank accession number NZ_ACQB00000000.1) deposited in GenBank by the Broad Institute as part of the Human Microbiome Project (http://www.broadinstitute.org/). Furthermore, attempts to identify the ATCC 19606^T^ *entA* gene by PCR amplification or Southern blotting, using ATCC 17978 genomic information to design appropriate primers and probes, failed to produce positive results (data not shown). Taken together, all these observations indicate that there are variations in the nucleotide sequence and chromosomal arrangements among the *entA* genes present in different *A. baumannii* strains.

**Table 2:** Revised annotation of the *A. baumannii* ATCC 17978 A1S_2562-A1S_2581 gene cluster.

Coding region #^a^	Annotated coding region^b^	Predicted function^c^
1	A1S_2562	MDR efflux pump protein
2	A1S_2563/A1S_2564	Siderophore interacting protein
3	A1S_2565	MFS family transport protein
4	A1S_2566	Ferric siderophore receptor protein
5	A1S_2567	Thioesterase
6	A1S_2568	Phosphopantetheinyl transferase
7	Omitted	Transposase fragment
8	A1S_2569	Transposase fragment
9	A1S_2570	Acetyltransferase
10	A1S_2571	Ornithine decarboxylase
11	A1S_2572	Lysine/ornithine monooxygenese
12	A1S_2573/A1S_2574	2,3 DHB-AMP ligase
13	A1S_2575	Nonribosomal peptide synthetase
14	A1S_2576/A1S_2577	Nonribosomal peptide synthetase
15	A1S_2578	Nonribosomal peptide synthetase
16	A1S_2579	2,3 DHB-2,3-dehydrogenase
17	A1S_2580	Isochorismatase
18	A1S_2581	Isochorismate synthetase

a Numbers correspond to coding regions shown in Fig. 1B.

b Coding regions reported by Smith et al. [39] and deposited in GenBank under accession number NC_009085.1.

c MDR, Multidrug Resistance; MFS, Major Facilitator Superfamily; DHB, dihydroxybenzoate.

### Cloning and testing of the ATCC 19606^T^ *entA* gene

**Figure 2:**
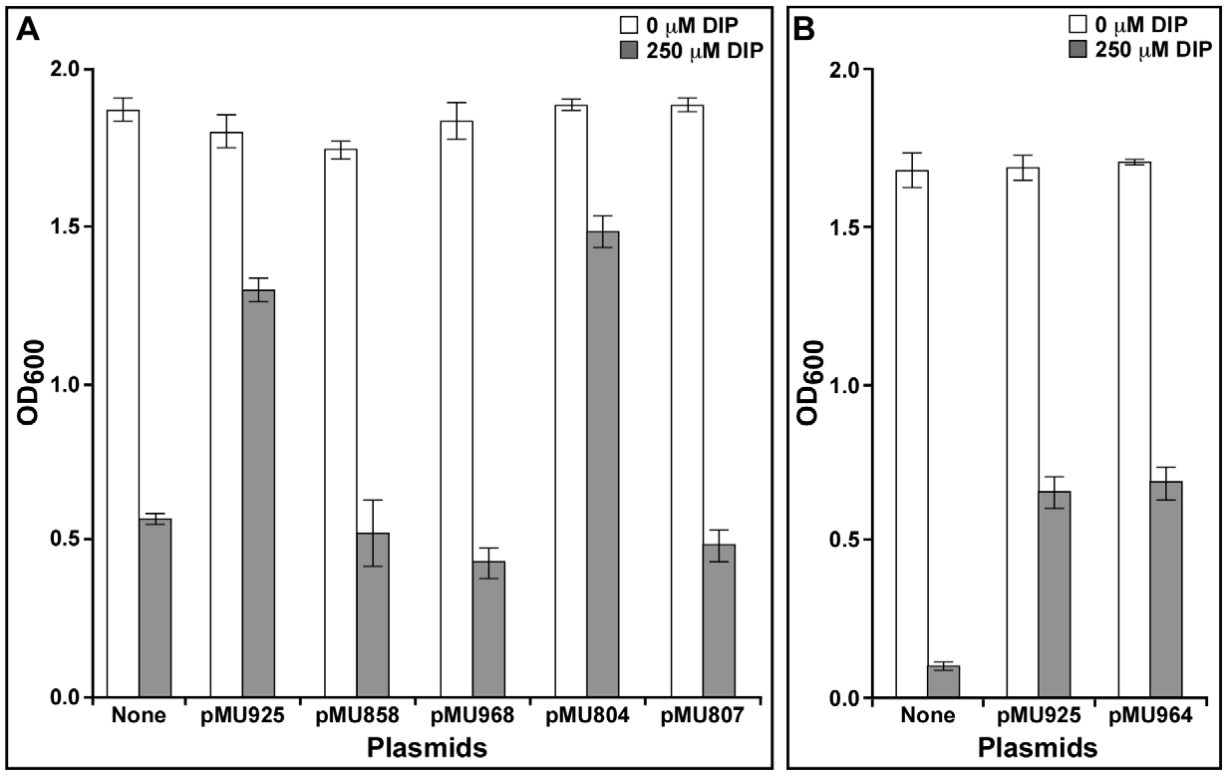
Iron acquisition phenotype of *E. coli* enterobactin deficient mutants. (A) Growth of the *E. coli* AN193 *entA* mutant harboring no plasmid or transformed with pMU925, pMU858, pMU968, pMU804 or pMU807 in LB broth in the absence or the presence of 250 µM DIP. (B) Growth of the *E. coli* AN192 *entB* mutant harboring no plasmid or transformed with pMU925 or pMU964 in LB broth in the absence or the presence of 250 µM DIP. The plasmids pMU925, pMU968 and pMU804 harbor the *entA*/*entB* orthologs cloned from ATCC 19606^T^, AYE, and ATCC 17978, respectively. Plasmid pMU858 harbors an insertionally inactivated ATCC 19606^T^ *entA* derivative. Plasmid pMU964 harbors the ATCC 19606^T^ *basF* gene.

Since the *in silico* approach failed to locate the ATCC 19606^T^ *entA* gene, a functional complementation approach was applied using *E. coli* AN193, an *entA* mutant that does not produce enterobactin because of its inability to make the DHBA precursor. Transformation of this mutant with plasmid DNA isolated *en masse* from an ATCC 19606^T^ genomic library made in *E. coli* LE392, using the cloning cosmid vector pVK100, resulted in the isolation of the AN193-2631 derivative capable of growing in the presence of significantly higher DIP concentrations when compared with AN193 transformed with empty cloning vector. Transformation of *E. coli* AN193 with plasmid pMU711 isolated from the AN193-2631 derivative confirmed the ability of this cosmid clone to restore the iron uptake capacity of *E. coli* AN193 (data not shown). Restriction analysis of pMU711 digested with HindIII showed that it has an insert larger than 20 kb (data not shown). Subcloning into HindIII-digested pUC118 and nucleotide sequencing resulted in the identification of pMU748, which has a 2.7-kb HindIII insert (Fig. 1A). This restriction fragment harbors a 771-nucleotide gene, the nucleotide sequence of which is identical to the ATCC 19606^T^ HMPREF0010_00620.1 locus found in the scaffold supercont 1.1 of the whole genome sequence uploaded to GenBank by the Broad Institute under accession number NZ_GG704572. This gene codes for a 28-kDa predicted protein highly related (E values lower than 1×e^−5^) to the EntA protein found in a wide range of bacteria, showing the top BLASTx scores with products of the cognate *A. baumannii* AB0057 (AB57_1983), AB307-294 (ABBFA_001741), and ACICU (ACICU_01790) genes (Table 3). Transformation of the *E. coli* AN193 *entA* mutant with either pMU748 (data not shown) or the PCR derivative pMU925, which includes a downstream *entB* ortholog (Fig. 1A), restored iron uptake proficiency to this derivative (Fig. 2A). The function of the ATCC 19606^T^ *entA* ortholog was confirmed by the observation that the AN193-3101 transformant harboring pMU858, a pUC118 HindIII subclone in which *entA* was inactivated by inserting a DNA cassette coding for Km resistance into a unique EcoRV site (Fig. 1A), showed a growth similar to that of untransformed *E. coli* AN 193 cells when cultured in the presence of 250 µM DIP (Fig. 2A).

**Table 3:** Components of the gene cluster containing the *entA-entB* genes in different *A. baumannii* genomes.

Strain	Annotated coding regions	Additional coding regions^a^	Accession #
19606^T^	00624.1-00618.1^b^	NA^c^	NZ_GG704572
AB0057	AB57_1980-AB57_1985	None	NC_011586.1
AB307-294	ABBFA_001744-ABBFA_001739	None	NC_011595.1
ACICU	ACICU_01787-ACICU_01792	None	NC_010611.1
AYE	AABAYE1887-ABAYE1894	ABAYE1890	NC_010410

a Additional coding regions compared to the ATCC 19606^T^ gene cluster shown in Fig. 1A.

b Numbers should be preceded by the annotation HMPREF0010_ according to the information presented in the *A. baumannii* Broad Institute genome site.

c Not applicable.

**Figure 3:**
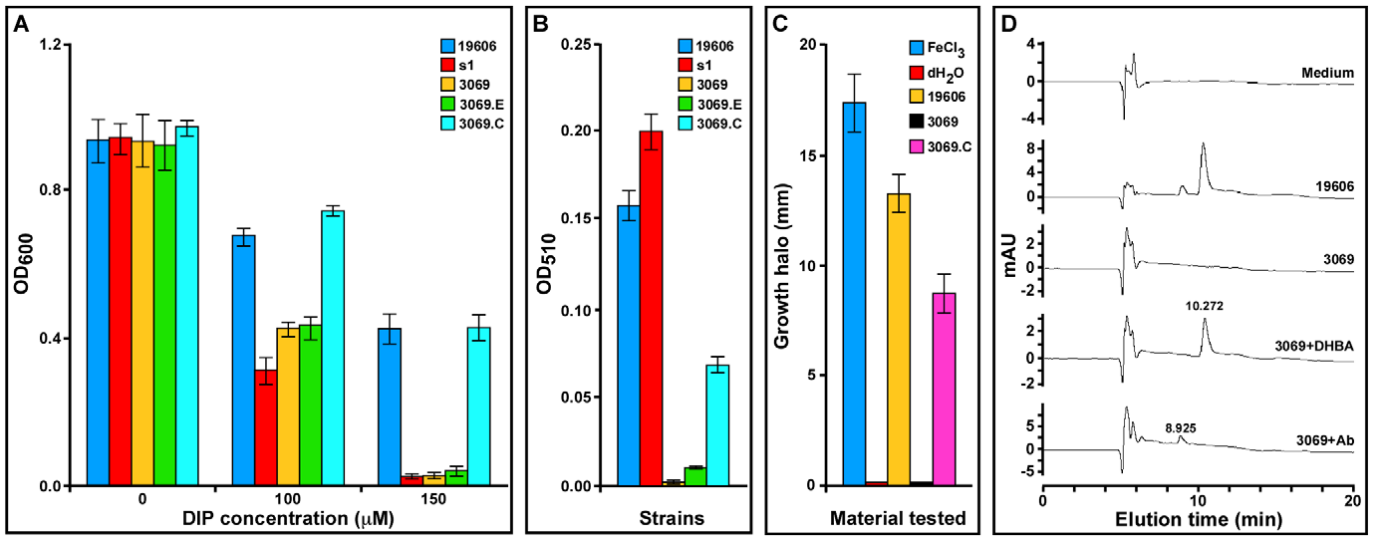
Iron acquisition phenotype of *A. baumannii* derivatives and their capacity to produce catechol, DHBA and acinetobactin. (A) The ATCC 19606^T^ parental strain (19606), the s1 BasD acinetobactin synthesis mutant (s1) and the 3069 *entA* isogenic derivative harboring no plasmid (3069) or transformed with empty pWH1266 vector (3069.E) or pMU951 harboring (3069.C) were cultured in M9 minimal medium in the absence or the presence of increasing concentrations of DIP. pMU951 is a pWH1266 derivative harboring the ATCC 19606^T^ *entA* allele. (B) Arnow's reaction of succinate culture supernatants obtained from the strains shown in panel A. (C) Crossfeeding of the *S. typhimurium enb*-7 enterobactin deficient derivative by FeCl_3_, distilled water or succinate culture supernatants from the ATCC 19606^T^ parental strain (19606) or the 3069 *entA* isogenic derivative harboring either no plasmid (3069) or pMU951 (3069.C) under iron-chelated conditions. (D) HPLC profiles of sterile succinate medium (medium) or culture supernatants from the ATCC 19606^T^ parental strain (19606) or the 3069 *entA* mutant (3069). Succinate culture supernatants cleared by high-speed centrifugation and filtration trough 0.45 µm cartridges were spiked with either DHBA (3069+DHBA) or purified acinetobactin (3069+Ab) immediately before HPLC analysis. The elution time for DHBA and acinetobactin are indicated on top of the cognate peaks.

**Figure 4:**
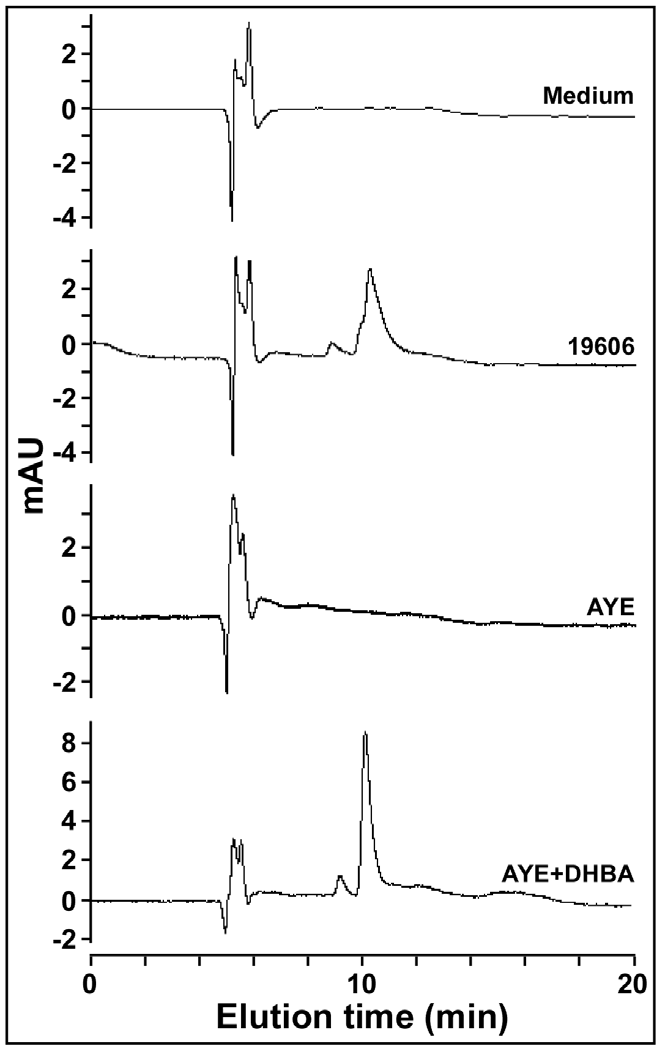
Analysis of culture supernatants. HPLC profiles of sterile succinate medium (medium) or culture supernatants from the ATCC 19606^T^ parental strain (19606) or the AYE clinical isolate cultured in the absence (AYE) or the presence of 100 µM DHBA (AYE+DHBA).

The role of the ATCC 19606^T^ *entA* ortholog in the acinetobactin-mediated iron acquisition process was confirmed further with the isogenic derivative 3069, which was generated by allelic exchange using pMU902 (Fig. 1A). This is a derivative of pEX100T, which does not replicate in ATCC 19606^T^, harboring the *entA*::*aph* construct. Compared with the ATCC 19606^T^ parental strain, the 3069 derivative, which showed the predicted genetic arrangement by PCR and Southern blotting (data not shown), displayed a drastic growth defect (*P* = 0.0001) when cultured in M9 minimal medium containing increasing DIP concentrations (Fig. 3A). This response is similar to that displayed by the ATCC 19606^T^ s1 mutant impaired in BasD-mediated acinetobactin biosynthesis activity [14], which was used as a control. The CAS colorimetric assay showed that while the ATCC 19606^T^ succinate culture supernatants tested positive, no reaction could be detected with culture supernatants of the ATCC 19606^T^ 3069 and s1 mutants (data not shown). Furthermore, Arnow colorimetric assays showed that 3069 cells produced drastically reduced amounts of DHBA (*P* = 0.002), which were within the detection limit of the Arnow test (Fig. 3B). This finding was supported by the lack of crossfeeding of the *S. typhimurium enb*-7 reporter mutant (Fig. 3C), which uses DHBA as a precursor to produce enterobactin and grow under iron-chelated conditions. Finally, HPLC analysis of culture supernatants of cells grown in succinate medium showed the presence of two peaks with elution times of 8.925 and 10.272 min (Fig. 3D). Although these two peaks were absent in the sterile medium as well as in the supernatant of the ATCC 19606^T^ 3069 mutant, they could be detected in the ATCC 19606^T^ 3069 sample only when it was spiked with either pure DHBA or purified acinetobactin before HPLC analysis (Fig. 3D). These observations showed that the two peaks detected in ATCC 19606^T^ culture supernatants indeed correspond to DHBA and mature acinetobactin. Interestingly, the chromatograms shown in Fig. 3D and Fig. 4 indicate that ATCC 19606^T^ cells produce and secrete a significant amount of DHBA in addition to fully matured acinetobactin. A similar analysis of s1 succinate culture supernatants showed the presence of DHBA but not fully matured acinetobactin (data not shown), a result that is in accordance with the colorimetric data shown in Fig. 3B.

Finally, the role of the ATCC 19606^T^ *entA* ortholog was confirmed with genetic complementation assays, which proved that electroporation of pMU951, a derivative of the shuttle vector pWH1266 harboring the *entA* wild-type allele expressed under the promoter controlling the expression of tetracycline resistance, was enough to restore the parental iron utilization phenotype in the ATCC 19606^T^ 3069.C derivative (Fig. 3A) as well as its capacity to produce DHBA (Fig. 3, panels B and C). This complementation was not observed with the ATCC 19606^T^ 3069.E derivative harboring the empty cloning vector pWH1266. Although not shown, there were no significant growth differences among the parental strain, the mutants and the complemented strains used in these experiments when they were cultured in LB broth without any selection pressure. Furthermore, the plasmid pMU951 was stably maintained in the ATCC 19606^T^ 3069.C strain as an independent replicon without detectable rearrangements.

Taken together, all these results strongly indicate that the *entA* ortholog shown in Fig. 1A is essential for the production of DHBA, which is used by *A. baumannii* ATCC 19606^T^ cells as a key precursor for acinetobactin biosynthesis. The location of the *entA* gene outside the main locus involved in the biosynthesis, transport and secretion of acinetobactin resembles the arrangement of genes needed for the biosynthesis of anguibactin in the fish pathogen *V. anguillarum* 775 [41]. The pJM1 plasmid present in this strain contains most of the genes coding for anguibactin biosynthesis and transport functions, including an *angA* ortholog that is inactive because of a frameshift mutation [42]. Further analysis showed that the 2,3-dihydro-2,3-dihydroxy-benzoate dehydrogenase activity needed for anguibactin biosynthesis is indeed encoded by the chromosomal ortholog *vabA*, which is located within a 11-gene cluster that contains all the genetic determinants needed for the production and utilization of vanchrobactin [43]. Although this system is fully active in *V. anguillarum* RV22, an O2 serotype isolate that lacks pJM1, vanchrobactin is not produced in the serotype O1 *V. anguillarum* 775 (pJM1) strain because of an RS1 transposon insertion within *vabF*. Furthermore, *in silico* analysis of the predicted product of *vabA* and *angA* loci, after virtual correction of the frameshift present in the later gene, showed that they are only 32% identical [41]. These observations and the fact that the *angA* pJM1 plasmid copy is near transposable elements led to the hypothesis that transposition events resulted in the acquisition by horizontal transfer of two genes potentially coding for the same function that may have evolved independently [41]. A mechanism similar to this one could also explain the location of the ATCC 19606^T^ *entA* gene outside the acinetobactin cluster [17], a possibility that is supported by the recent observation that *A. baumannii* has an underappreciated capacity to rearrange its genome by swapping, acquiring or deleting genes coding for a wide range of functions, including those involved in iron acquisition [44].

Our results also provide strong support to our previous report that acinetobactin is the only high-affinity siderophore produced by this strain [14], when cultured under iron-chelated laboratory conditions, although it contains genetic determinants that could code for additional iron acquisition functions as deduced from recent comparative genomic analyses [16], [17]. Considering these reports and the experimental data presented here, it is possible to speculate that the additional iron-acquisition related genes present in *A. baumannii* ATCC 19606^T^ are either not expressed or do not code for complete functional iron acquisition systems and represent remnants of DNA fragments acquired from other sources by horizontal gene transfer. Such outcomes, which are currently being explored, could be due to a situation similar to that reported for the fish pathogen *V. anguillarum*, where a vanchrobactin-producing ancestor acquired the pJM1 plasmid coding for the production and utilization of anguibactin [43]. Since anguibactin is potentially a better iron chelator than vanchrobactin, evolution and adaptation processes favored the emergence of *V. anguillarum* O1 serotype strains harboring the pJM1 plasmid and producing only anguibactin because of mutations in the vanchrobactin coding genes, but capable of using both anguibactin and vanchrobactin as it is the case with the 775 (pMJ1) strain [45].

### Analysis of the ATCC 19606^T^ chromosomal region harboring *entA* and *entB* orthologs

Sequence analysis of flanking DNA regions showed that a *modE*-*modA-modB-modC* gene cluster, which could code for an uncharacterized molybdenum transport system, locates upstream of ATCC 19606^T^ *entA* gene (Fig. 1A). Downstream, *entA* is separated by a 72-nt intergenic region from a predicted gene coding for an isochorismatase (EntB) ortholog, which is followed by a 524-nt intergenic region preceding a *fur* iron-dependent regulatory gene. The nucleotide sequence and genetic arrangement is the same as that reported for this strain by the Broad Institute Human Microbiome Project. Table 3 shows that the same gene cluster is also found in the genome of *A. baumannii* AB0057, AB307-294 and ACICU.

The predicted product of the ATCC 19606^T^ *entB* gene depicted in Fig. 1A showed significant amino acid sequence similarity and the same length when compared with the products of the cognate AB0057 (AB57_1984), AB307-294 (ABBFA_001740), ACICU (ACICU_01791), and AYE (ABAYE1888) genes. The product of the ATCC 19606^T^ *entB* ortholog is also related to that of *basF*, which is located within the acinetobactin gene cluster originally described in this strain [18]. The product of *basF* is a 289-amino acid protein that contains the N-terminal isochorismatase lyase (ICL) domain, which is needed for DHBA biosynthesis from isochorismic acid, and the C-terminal aryl carrier (ArCP) protein domain, which is needed for tethering activated DHBA and chain elongation in the biosynthesis of siderophores such as enterobactin [46] and anguibactin [47]. In contrast, *in silico* analysis showed that the *entB* ortholog shown in Fig. 1A codes for a 213-amino acid residue protein that includes the N-terminal ICL domain but lacks the C-terminal ArCP protein domain. Based on this predicted protein structure, it is possible to speculate that this shorter EntB ortholog should code for the isochorismatase lyase activity. This possibility was examined by testing the iron-uptake proficiency of the *E. coli* AN192 *entB* mutant harboring either no plasmid or the derivatives AN192-3171 and AN192-3170 transformed with pMU925, which harbors the *entA* and *entB* genes shown in Fig. 1A, or pMU964, which harbors a copy of the *basF* gene present in the acinetobactin cluster, respectively. Fig. 2B shows that AN192-3171 (pMU925) and AN192-3170 (pMU964) grew significantly better (*P*<0.0001 and *P* = 0.0013, respectively) than the non-transformed AN192 strain when cultured in LB broth containing 250 µM DIP. This response indicates that both ATCC 19606^T^ *entB* orthologs complement the *E. coli* AN1932 *entB* mutant, allowing it to produce enterobactin and grow under iron-chelated conditions. The complementation by the BasF ortholog is straightforward since this protein contains the ICL and ArCP protein domains needed for enterobactin biosynthesis. On the other hand, the complementation by the shorter ATCC 19606^T^ EntB ortholog can be explained by the previous observation that the mutation in *E. coli* AN192 affects only the functionality of the ICL domain of the protein produced by this derivative that was obtained by chemical mutagenesis [31]. This functional observation is further supported by our DNA sequencing data showing that the AN192 *entB* ortholog has two point mutations when compared with the parental AB1515 allele. One is a silent mutation that resulted in a G-to-A base transition at position 801 of the gene, which maps within the ArCP protein domain and produced no change in amino acid sequence. In contrast, the other mutation, which locates at position 593 and is also a G-to-A base transition, produced a Gly-to-Asp amino acid change at position 198 that maps within the ICL domain. This amino acid change, which is one residue away from Arg196 that is predicted to play a role in the interaction of the ICL domains in the functional EntB dimer protein [48], could be responsible for the lack of isochorismatase lyase in the AN192 mutant. Taken together, these results indicate that *basF*, which locates within the acinetobactin gene cluster, is the only fully functional *entB* ortholog present in the genome of the *A. baumannii* strains fully sequenced and annotated.

Considering the different genetic context in which *basF* and the *entB* ortholog shown in Fig. 1A are found in different *A. baumannii* genomes and the differences in length (289 vs. 213 amino acid residues) and amino acid composition (52.4% similarity and 40.1% identity to one another) of their predicted products, it is possible to speculate that these two genes were transferred from unrelated sources by at least two independent horizontal mechanisms, one of which may have been driven by the need of acquiring the *entA* trait required for acinetobactin biosynthesis. These observations are in agreement with the recently described capacity of *A. baumannii* to rearrange its genome content [44], as proposed above for the presence of the *entA* ortholog in a genomic region outside the acinetobactin cluster.

### Analysis of the *A. baumannii* AYE *entA* ortholog

The comparative genomic analysis of the ATCC 19606^T^ *entA* ortholog with other *A. baumannii* sequenced genomes showed that the clinical isolate AYE also harbors the gene cluster shown in Fig. 1A, which was annotated as ABAYE1887-ABAYE1894 [36]. However, this cluster contains an additional 210-nucleotide gene (ABAYE1890) (Table 3), which was not included in any of the other fully sequenced *A. baumannii* genomes. ABAYE1890 codes for a hypothetical 69-amino acid protein and overlaps by 35 nucleotides with ABAYE1889, which codes for a predicted 229-amino acid protein. Both genes were recently recognized by comparative genomic analysis as components of the siderophore biosynthesis gene cluster 5 [17]. However, it is important to note that the predicted product of ABAYE1889 is 26 amino acids shorter than the putative ACICU, AB0057 and AB307-0294 EntA orthologs. This observation suggests that the *A. baumannii* AYE ABAYE1889 *entA* gene may not code for a functional product needed for the biosynthesis of DHBA and acinetobactin. This possibility was supported by the HPLC analysis of AYE succinate culture supernatants, which showed an elution profile that does not include peaks corresponding to DHBA and acinetobactin that were detected in the ATCC 19606^T^ culture supernatant (Fig. 4). Interestingly, AYE succinate culture supernatants promoted the growth of *S. typhimurium enb*-7 in the presence of DIP in spite of the fact that it does not produce DHBA (data not shown). This observation indicates that *A. baumannii* AYE produces an uncharacterized non-catechol-based siderophore(s) capable of promoting growth under iron-chelated conditions.

The failure of the ABAYE1889-ABAYE1890 genetic region to code for EntA activity was further confirmed by the fact that AN193-3179, a derivative transformed with pMU968 harboring the 2.8-kb AYE chromosomal region encompassing these predicted genes, grew as poorly as the non-complemented AN193 mutant when cultured in the presence of 250 µM DIP (Fig. 2A). All these results indicate that the AYE isolate is a natural *A. baumannii entA* mutant that does not make acinetobactin because of the lack of DHBA production. HPLC analysis of AYE culture supernatants of cells grown in succinate medium supplemented with 100 µM DHBA confirmed this possibility since a peak with a retention time corresponding to acinetobactin could be detected only under this experimental condition (Fig. 4).

Cloning, DNA sequencing and bioinformatic analysis of the 2.8-kb AYE chromosomal region encompassing the ABAYE1889 and ABAYE1890 genes confirmed the original genome sequence report [36] and showed that the only difference between this region and the cognate regions of other *A. baumannii* chromosomes is the presence of an extra T at position 1,946,969 in the AYE genome. This single-base insertion, which could be due to DNA slippage, results in the two predicted annotated genes, neither of which code for a full-length EntA ortholog. Accordingly, *in silico* deletion of the extra T from the AYE genomic region results in a single predicted coding region, the product of which is an ortholog displaying the same number of amino acid residues predicted for the product of the ATCC 19606^T^ *entA* gene shown in Fig. 1A. All these results indicate that the AYE clinical isolate is a natural *entA* mutant incapable of producing acinetobactin, although this isolate tests positive with the CAS reagent. This situation could be similar to that of *V. anguillarum* 775 (pJM1) that only produces anguibactin but uses this siderophore as well as externally provided vanchrobactin to acquire iron under chelated conditions [43]. This is due to the inactivation of the *vabF* vanchrobactin chromosomal gene and the possibility that anguibactin is an iron chelator stronger than vanchrobactin, a condition that does not justify the production of two siderophores. Accordingly, the production and utilization of the AYE uncharacterized siderophore, which may have a higher affinity for iron than acinetobactin, could be mediated by genes located in cluster 1 identified by *in silico* genomic analysis [17] that remain to be characterized genetically and functionally. Furthermore, preliminary siderophore utilization bioassays showed that AYE cell-free succinate culture supernatants crossfeed the ATCC 19606^T^ s1 (*basD*) and 3069 (*entA*) acinetobactin production deficient mutants as well as the t6 (*bauA*) acinetobactin uptake mutant. These findings indicate that *A. baumannii* AYE acquires iron via an uncharacterized siderophore, which is different from acinetobactin but can be used by ATCC 19606^T^ cells via acinetobactin-independent mechanisms. All these results provide evidence supporting the ability of the ATCC 19606^T^ strain to use xenosiderophores produced by related and unrelated bacterial pathogens.

### Analysis of the ATCC 17978 chromosomal region harboring the *entA* ortholog

The ATCC 17978 A1S_2562-A1S_2581 cluster (Table 2), cluster 2 according to Eijkelkamp *et al.* [17], includes the A1S_2579 gene coding for a putative EntA ortholog [39]. The role of this gene in DHBA production was confirmed by the observation that the AN193-2943 transformant harboring pMU804 (Fig. 1B) displays an iron-restricted response similar to that detected with AN193-3172 harboring pMU925, a response that was not detected with AN193-2944 harboring pMU807 (Fig. 2A). The latter plasmid is a derivative of pMU804 with a transposon insertion within the annotated *entA* (A1S_2579) coding region (Fig. 1B).

Detailed analysis of the nucleotide sequence and annotation of the A1S_2562-A1S_2581 cluster (GenBank accession number NC_009085.1) showed that because of potential DNA sequencing errors, this cluster is most likely composed of 18 predicted coding regions rather than the 20 genes originally reported [39]. Fig. 1B and Table 2 show that A1S_2563 and A1S_2564 could be a single genetic unit coding for a predicted siderophore interacting protein that belongs to the ferrodoxin reductase protein family. Our DNA sequencing data, which confirmed the DNA sequence originally reported [39], and *in silico* analysis showed that there are two potential coding regions between A1S_2568 and A1S_2570 (Fig. 1B and Table 1), with one of them annotated as the A1S_2569 coding region and the other omitted in the original report. Our analysis showed that the predicted products of these two genes are truncated transposases found in a wide range of bacterial genomes including members of the *Acinetobacter* genus. We also observed that A1S_2573 and A1S_2574 most likely correspond to a single genetic unit coding for a predicted 2,3 dihydroxybenzoate-AMP ligase (EntE) (Fig. 1B and Table 1), which is needed for the activation of DHBA and further biosynthesis of DHBA-containing siderophores such as enterobactin and anguibactin [10]. Nucleotide and amino acid comparative analyses also showed that the products of A1S_2573/A1S_2574, A1S_2580 and A1S_2581 are significantly related to that of the *basE*, *basF* and *basJ* genes, respectively. The products of these three genes, which work together with EntA, are needed for the biosynthesis and activation of DHBA using chorismate as a precursor. Taken together, all these observations indicate that there is a potential redundancy in the *A. baumannii* ATCC 17978 functions needed for the biosynthesis and utilization of DHBA as a siderophore precursor. In contrast, and as it was observed with the other *A. baumannii* genomes, the ATCC 17978 *entA* ortholog, which is represented by the coding region A1S_2579 (Fig. 1B and Table 1), is a unique coding region that locates outside the *bas-bau-bar* gene cluster. Furthermore, the genetic arrangement and content of this ATCC 17978 gene cluster containing the *entA* ortholog is different from that shown in Fig. 1A and described in the genome of other *A. baumannii* strains. This observation resulted in the classification of this cluster as cluster 2 [17], which at the time that report was published was found only in the ATCC 17978 genome. However, genomic data recently uploaded into the BaumannoScope web site (https://www.genoscope.cns.fr/agc/microscope/about/collabprojects.php?P_id=8) indicate that the same gene cluster is also present in the genome of the *A. baumannii* strains 6013113 (GenBank accession number ACYR02000000.2) and 6013150 (GenBank accession number ACYQ00000000.2).

Interestingly, the ATCC 17978 A1S_2562-A1S_2581 gene cluster has the same genetic content and organization as that of the *A. baylyi* ADP1 ACIAD2761-ACIAD2776 gene cluster (GenBank accession number NC_005966.1) with the exception of the presence of the transposase coding regions. A gene potentially coding for a transposase fragment is located outside the *A. baylyi* ADP1 ACIAD2761-ACIAD2776 gene cluster, downstream of ACIAD2761 [49]. In contrast, the ATCC 17978 cluster 2 includes two putative genes coding for transposase-related proteins located between the A1S_2568 and A1S_2570 annotated coding regions (Fig. 1B and Table 1), one of which was not included in the original report [39]. Furthermore, our previous analysis showed that cluster 2 includes perfect inverted repeats located at the ends of the cluster [40]. Taken together, these observations suggest that this particular cluster was mobilized by horizontal gene transfer among environmental and clinical *Acinetobacter* strains, which must acquire iron in different free-iron restricted ecological niches.

### Role of the ATCC 19606^T^ *entA* gene in virulence

**Figure 5:**
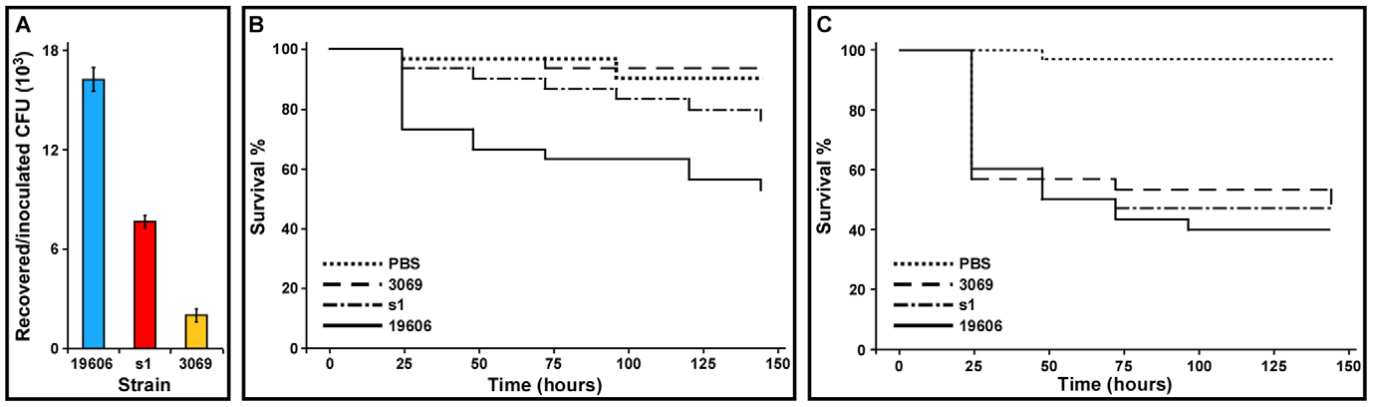
Virulence of *A. baumannii* ATCC 19606^T^ parental and isogenic derivatives. (A) Persistence of bacteria in the presence of A549 monolayers. Bacteria (10^3^) we added to 95% confluent monolayer maintained in mHBSS. The resulting CFUs were quantified after 24 h incubation at 37°C in 5% CO_2_. (B and C) *G. mellonella* killing assays. Caterpillars were infected with 1×10^5^ bacteria of the ATCC 19606^T^ parental strain (19606), or the s1 or 3069 iron-deficient isogenic derivatives in the absence (panel B) or the presence (panel C) of 100 µM Fe_3_Cl and incubated at 37°C in darkness. Moth death was determined daily for six days. Caterpillars injected with comparable volumes of PBS or PBS plus 100 µM Fe_3_Cl were used as negative controls.

The role of the *entA* gene in the virulence of *A. baumannii* ATCC 19606^T^ was tested using A549 human alveolar epithelial cells and *G. mellonella* caterpillars as *ex vivo* and *in vivo* experimental models. The tissue culture assays showed that the number of ATCC 19606^T^ bacteria recovered after 24 h incubation was significantly greater than the s1 (*P* = 0.0048) and 3069 (*P* = 0.000045) derivatives (Fig. 5A), affected in acinetobactin biosynthesis at intermediate (*basD*) and early (*entA*) biosynthetic stages, respectively. It was also noted that the persistence of 3069 was significantly lower than that of the s1 mutant (*P* = 0.0024) (Fig. 5A). It is important to mention that incubation of *A. baumannii* ATCC 19606^T^ in mHBSS at 37°C for 24 h did not result in significant bacterial growth.

Infection of *G. mellonella* larvae showed that more than 40% of them died six days after injected with the parental ATCC 19606^T^ strain, a value that is significantly different (*P* = 0.0014) from that obtained with animals injected with sterile PBS (Fig. 5B). Infection of caterpillars with the 3069 *entA* mutant showed that the killing rate of this derivative is not statistically different from that of the PBS negative control (*P* = 0.6705) but significantly different from the killing rate of the parental strain (*P* = 0.0005). On the other hand, the s1 derivative is significantly more virulent than the 3069 mutant (*P* = 0.043) and almost significantly different from the parental strain (*P* = 0.0820). The killing rates of the s1 and 3069 mutants were corrected to values statistically indistinguishable from the parental strain when the inocula used to infect the larvae were supplemented with 100 µM FeCl_3_ (Fig. 5C). This response shows that the virulence defect of the s1 and 3069 isogenic mutants is due to their inability to acquire iron when injected into *G. mellonella*.

Taken together, these results demonstrate that inactivation of *entA* produces a more drastic reduction in the capacity of *A. baumannii* ATCC 19606^T^ cells to persist in the presence of A549 cells, which represent a target affected during the respiratory infections this pathogen causes in humans, or infect and kill *G. mellonella* larvae, an invertebrate host capable of mounting a complex innate immune response similar to that of vertebrate animals [50], when compared to the response obtained with the *basD* mutant. This response could be due to the fact that the 3069 derivative does not produce DHBA and acinetobactin intermediates, which are secreted by the s1 mutant and could have some functions in iron acquisition and virulence as it was shown with *Brucella abortus* [51], [52]. Thus, these acinetobactin precursors together with DHBA could bind iron, although less efficiently than acinetobactin, and provide s1 cells with enough iron to persist and cause host injury, although to a much reduced extent when compared with the response obtained with ATCC 19606^T^ cells.

### Conclusions

The work described in this report shows that a single *A. baumannii entA* functional ortholog, which is essential for the biosynthesis of the acinetobactin precursor DHBA, is located outside the acinetobactin gene cluster, which otherwise codes for all biosynthesis, secretion and transport functions related to iron acquisition mediated by this high-affinity siderophore. Although the same genetic arrangement is found in all fully sequenced and annotated *A. baumannii* genomes, the genetic context within which the *entA* ortholog is found varies among different clinical isolates. In one group of strains this gene is next to genes coding for putative molybdenum transport, as in ATCC 19606^T^. In another group, *entA* is located within a large gene cluster, which could code for an alternative uncharacterized siderophore-mediated system, as in ATCC 17978. Interestingly, this cluster also includes genes coding for potential DNA mobility functions and is flanked by perfect inverted repeats, a feature that may explain its transfer by lateral processes. Nevertheless, in all cases examined the *entA* ortholog is always next to an *entB* ortholog, which codes for a protein that is able to catalyze the production of DHBA from isochorismic acid but cannot promote the completion of the acinetobactin biosynthesis process because of the lack of the C-terminal aryl carrier (ArCP) protein domain. This is in contrast to the presence of *basF* within the acinetobactin cluster that codes for a fully functional EntB ortholog. All these findings indicate that all genetic components needed for iron acquisition via the acinetobactin-mediated system involved horizontal transfer as well as complex chromosomal rearrangement processes. All these observations are in agreement with the recent observation that *A. baumannii* has the capacity to acquire, lose and/or shuffle genes, a number of which could code for virulence factors involved in the pathogenesis of the infections this bacterium causes in humans [44]. Our study also shows that the presence of all genetic determinants needed for the biosynthesis and utilization of acinetobactin does not warrant its active expression and may not reflect a virulence advantage when compared to other strains; the AYE clinical isolate is a natural *entA* mutant incapable of producing DHBA and acinetobactin. However, AYE is an iron-uptake proficient strain that seems to acquire this essential metal due to the expression of a siderophore-mediated system that remains to be functionally characterized. This finding reinforces our initial observation that different *A. baumannii* clinical isolates can express different iron acquisition systems [12]. Finally, we show that acinetobactin intermediates and DHBA, which are produced in addition to acinetobactin in the ATCC 19606^T^ strain, play a role in the virulence of *A. baumannii* when tested using *ex vivo* and *in vivo* infection experimental models, although to a lesser extent when compared to the role of the fully matured acinetobactin-mediated system.

## Supporting Information

Table S1 Bacterial strains and plasmids used in this work.
(DOC)
