# Supplementary material for: The Acinetobacter baumannii entA Gene Located Outside the Acinetobactin Cluster Is Critical for Siderophore Production, Iron Acquisition and Virulence
Source: PLoS One. 2012 May 3;7(5):e36493. doi: 10.1371/journal.pone.0036493 (PMC3343012; doi:10.1371/journal.pone.0036493)
Supplement: Table S1 — Bacterial strains and plasmids used in this work. (DOC) [file pone.0036493.s001.doc]

**Table S1.** Bacterial strains and plasmids used in this work.

| Strain/plasmid | Relevant characteristics^a^ | Source/reference |
| --- | --- | --- |
| Strains |  |  |
| *A. baumannii* |  |  |
| ATCC 19606^T^ | Nosocomial isolate | ATCC |
| ATCC 19606^T^ s1 | ATCC 19606^T^ *basD* mutant, Km^R^ | [1] |
| ATCC 19606^T^ 3069 | ATCC 19606^T^ *entA* mutant, Km^R^ | This work |
| ATCC 19606^T^ 3069.C | ATCC 19606^T^ 3069 harboring pMU951 | This work |
| ATCC 19606^T^ 3069.E | ATCC 19606^T^ 3069 harboring pWH1266 | This work |
| ATCC 17978 | Nosocomial isolate | ATCC |
| AYE | Nosocomial isolate | [2] |
| *E. coli* |  |  |
| LE392 | Used for *in vitro* DNA packaging | [3] |
| DH5α | Used for recombinant DNA methods | Gibco-BRL |
| AB1515 | *purE42*, *proC14*, *leu-6*, *trpE38*, *thi-1*, *fhuA23*, *lacYl*, *mtl-l*, *xyl-5*, *rpsL109*, *azi-6*, *tsx-67* | CGSC^b^ |
| AN192 | *entB* mutant of AB1515 | [4] |
| AN192-3170 | AN192 harboring pMU964 | This work |
| AN192-3171 | AN192 harboring pMU925 | This work |
| AN193 | *entA* mutant | C. Earhart |
| AN193-2631 | AN193 harboring pMU711 | This work |
| AN193-2942 | AN193 harboring pMU748 | This work |
| AN193-2943 | AN193 harboring pMU804 | This work |
| AN193-2944 | AN193 harboring pMU807 | This work |
| AN193-3101 | AN193 harboring pMU858 | This work |
| AN193-3172 | AN193 harboring pMU925 | This work |
| AN193-3179 | AN193 harboring pMU968 | This work |
| *S. typhimurium* |  |  |
| *enb*-7 | Uses DHBA to produce enterobactin and grow under iron-chelated conditions | [5] |
| Plasmids |  |  |
| pVK100 | Cosmid cloning vector, Tet^R^, Km^R^ | [6] |
| pUC118 | Cloning vector, HindIII/BAP, Amp^R^ | Takara |
| pUC4K | Source of Km^R^ DNA cassette, Amp^R^, Km^R^ | Pharmacia |
| PCR-Blunt | PCR cloning vector, Km^R^, Zeo^R^ | Invitrogen |
| PCR8/GW/TOPO | PCR cloning vector, Sp^R^ | Invitrogen |
| pEX100T | Mobilizable suicide plasmid in ATCC 19606^T^, Amp^R^ | ATCC |
| pWH1266 | *E. coli*-*A. baumannii* shuttle cloning vector,  Amp^R^, Tet^R^ | [7] |
| pMU711 | pVK100 harboring the 19606^T^ *entA* gene,  Tet^R^, Km^R^ | This work |
| pMU748 | pUC118 with a 2.7-kb HindIII fragment from pMU711 harboring *entA*, *modB*, *modC* and the 3’end of *modA*, Amp^R^ | This work |
| pMU804 | PCR8/GW/TOPO harboring the ATCC 17978 *entA* and *entB* genes, Sp^R^ | This work |
| pMU807 | *entA*::EZ-Tn*5*<KAN-2> derivative of pMU804, Sp^R^, Km^R^ | This work |
| pMU858 | pMU748 derivative with ATCC 19606^T^ *entA*::*aph* insertion, Amp^R^, Km^R^ | This work |
| pMU902 | pEX100T with a 1.2 kb amplicon harboring *entA*::*aph,* Amp^R^, Km^R^ | This work |
| pMU925 | PCR-Blunt derivative harboring the ATCC 19606^T^ *entA* and *entB* genes, Km^R^ | This work |
| pMU951 | pWH1266 harboring the ATCC 19606^T^ *entA* allele, Amp^R^ | This work |
| pMU964 | PCR-Blunt derivative harboring the ATCC 19606^T^ *basF* gene, Km^R^ | This work |
| pMU968 | PCR-Blunt derivative harboring the AYE chromosomal region encompassing the predicted *entA*-*entB* orthologs, Km^R^ | This work |

^a^Amp, ampicillin; Km, kanamycin; Sp, spectinomycin; Tet, tetracycline; Zeo, zeocin; R, resistance/resistant. ^b^CGSC, Coli Genetic Stock Center, Yale University, New Haven, Conn.

**References**

1. Dorsey CW, Tomaras AP, Connerly PL, Tolmasky ME, Crosa JH, et al. (2004) The siderophore-mediated iron acquisition systems of *Acinetobacter baumannii* ATCC 19606 and *Vibrio anguillarum* 775 are structurally and functionally related. Microbiology 150: 3657-3667.

2. Vallenet D, Nordmann P, Barbe V, Poirel L, Mangenot S, et al. (2008) Comparative analysis of *Acinetobacters*: three genomes for three lifestyles. PLoS ONE 3: e1805.

3. Murray NE, Brammar WJ, Murray K (1977) Lambdoid phages that simplify the recovery of *in vitro* recombinants. Mol Gen Genet 150: 53-61.

4. Staab JF, Earhart CF (1990) EntG activity of *Escherichia coli* enterobactin synthetase. J Bacteriol 172: 6403-6410.

5. Pollack JR, Ames BN, Neilands JB (1970) Iron transport in *Salmonella typhimurium*: mutants blocked in the biosynthesis of enterobactin. J Bacteriol 104: 635-639.

6. Knauf VC, Nester EW (1982) Wide host range cloning vectors: a cosmid clone bank of an *Agrobacterium* Ti plasmid. Plasmid 8: 45-54.

7. Hunger M, Schmucker R, Kishan V, Hillen W (1990) Analysis and nucleotide sequence of an origin of DNA replication in *Acinetobacter calcoaceticus* and its use for *Escherichia coli* shuttle plasmids. Gene 87: 45-51.
